# Treatment with (chemo)-radiation in old patients (≥76 years of age) with newly diagnosed non-metastatic squamous cell cancer of the head and neck region: real-world data from a tertiary referral center

**DOI:** 10.3389/fonc.2024.1382405

**Published:** 2024-04-25

**Authors:** Leah-Sophie Winkler, Marlen Haderlein, Sabine Semrau, Florian Putz, Daniel Höfler, Sarina K. Müller, Heinrich Iro, Marco Kesting, Rainer Fietkau, Philipp Schubert

**Affiliations:** ^1^ Department of Radiation Oncology, University Hospital of Erlangen, Friedrich-Alexander-University Erlangen-Nürnberg (FAU), Erlangen, Germany; ^2^ Comprehensive Cancer Center Erlangen-Europäische Metropolregion Nürnberg (EMN), Erlangen, Germany; ^3^ Department of Otorhinolaryngology, Head and Neck Surgery, University Hospital of Erlangen, Friedrich-Alexander University Erlangen-Nürnberg (FAU), Erlangen, Germany; ^4^ Department for Oral and Maxillofacial Surgery, University Hospital Erlangen, Friedrich-Alexander-University Erlangen-Nürnberg, Erlangen, Germany

**Keywords:** head and neck cancer, squamous cell carcinoma, old patients, chemoradiation, tumor localization

## Abstract

**Purpose:**

Treatment of patients with cancer of the head and neck region is in focus in a multitude of studies. Of these patients, one patient group, those aged 76 and more, is mostly underrepresented despite requiring thorough and well-reasoned treatment decisions to offer curative treatment. This study investigates real-world data on curative treatment of old (≥76 years) patients with newly diagnosed squamous cell carcinoma of the head and neck region (HNSCC).

**Patients and methods:**

Between January 2010 and December 2021, we identified 71 patients older than 76 years with newly diagnosed HNSCC and cM0 at the Department of Radiation Oncology of the University Hospital of Erlangen-Nuremberg. Using electronic medical records, we analyzed treatment patterns and outcomes in terms of overall survival (OS), progression-free survival (PFS), and locoregional control (LRC) rate. Additionally, we performed univariate risk analysis and Cox regression in order to identify predictive factors associated with the abovementioned treatment outcomes.

**Results:**

The median follow-up was 18 months. OS was 83%, 79%, and 72% after 1 year, 2 years, and 3 years, respectively. PFS was 69%, 54%, and 46% after 1 year, 2 years, and 3 years, respectively. A total of 34 (48%) patients were treated with standard therapy according to current guidelines. The reasons for deviation from standard therapy before or during treatment were as follows: unfitness for cisplatin-based chemotherapy (n = 37), reduction of chemotherapy (n = 3), and dose reduction/interruption of radiotherapy (n = 8). Carboplatin-based systemic therapy showed improved PFS compared to cisplatin or cetuximab (60 *vs.* 28 *vs.* 15 months, p = 0.037) but without impact on OS (83 *vs.* 52 *vs.* 38 months, p = 0.807). Oropharyngeal tumor localization (p = 0.026) and combined treatment (surgery and postoperative treatment) (p = 0.008) were significant predictors for a better OS. In multivariate analysis, oropharyngeal tumor localization (p = 0.011) and combined treatment (p = 0.041) showed significantly increased PFS. After 1 year, 2 years, and 3 years, the cumulative incidence of locoregional recurrences (LRRs) was 13%, 24%, and 27%, respectively, and was significantly decreased in patients with oropharyngeal tumor localization (p = 0.037).

**Conclusions:**

Adherence to treatment protocols for radiotherapy alone in old patients with HNSCC is good, whereas the application of concurrent chemotherapy often deviates from guidelines in terms of de-escalation. An important risk factor for decreased OS, PFS, and a higher rate of LRR appears to be non-oropharyngeal tumor location in old patients.

## 1 Introduction

In a consensus panel, in patients diagnosed with squamous cell carcinoma of the head and neck region (HNSCC), those aged 61 to 75 years are classified as younger old, those aged 76 to 90 years as older old, and those older than 90 years as oldest old. There is a consensus that treatment outcomes likely do not correlate with chronological age but rather with biological age and comorbidities (1).

Head and neck carcinomas are the seventh most common cancer worldwide, accounting for 900,000 new cases/year and approximately 460,000 deaths/year (2, 3). As life expectancy increases, the number of older patients also rises (4). To date, the elderly are underrepresented in landmark prospective studies, which serve as the basis for the current gold standard in treating HNSCC patients. Consequently, there is a significant lack of information on how to treat old patients with newly diagnosed HNSCC, despite the need for thorough and highly individualized treatment strategies (4). This is particularly important considering that old patients are more likely to receive palliative treatment than curative treatment (5).

In this monocentric retrospective analysis, we describe treatment patterns of patients with HNSCC aged 76 years and older undergoing radio- and chemoradiation. Furthermore, we investigated treatment protocols (adherence to guidelines) and the feasibility of treatment. Additionally, we analyzed survival outcomes and prognostic factors.

## 2 Materials and methods

### 2.1 Data collection and patient characteristics

Electronic patient records were evaluated for patients aged 76 and older with newly diagnosed squamous cell carcinoma of the oral cavity, oropharynx, larynx, and hypopharynx treated in the Department of Radiation Oncology of the University Hospital of Erlangen-Nuremberg between January 1, 2010, and December 31, 2021. Exclusion criteria were recurrent tumors and the presence of distant metastases at the time of first diagnosis. A total of 71 patients met the inclusion criteria. TNM classification was according to the American Joint Committee on Cancer (AJCC) TNM seventh edition. Detailed information about patient characteristics is summarized in **Table 1** and **Supplementary Table 1**.

**Table 1:** Patient characteristics.

Characteristics	No. of patients	%
Sex		
Male Female	50 21	70.4 29.6
Age at radiotherapy, yrs		
Median Range	79 76 - 92	
Barthel Index		
Total dependency (0-30pts) Dependency (35-85) Partial dependency (85-95) Totally independent (100pts) No information	1 11 4 41 14	1.4 15.5 5.6 57.7 19.7
ECOG Performance status		
Asymptomatic (0) Symptomatic but completely ambulatory (1) Symptomatic, <50% in bed during the day (2) Symptomatic, >50% in bed, not bedbound (3) Bedbound (4) Death (5) Not defined	22 15 19 13 0 0 2	31.0 21.1 26.8 18.3 0 0 2.8
Charlson-Comorbidity Index		
1-year mortality rate 12% (0 pts) 1-year mortality rate 26% (1-2 pts) 1-year mortality rate 52% (2-4 pts) 1-year mortality rate 85% (>5 pts)	0 0 36 35	0 0 50.7 49.3
Primary tumor site		
Oral cavity Oropharynx Base of tongue Tonsil Uvula Palate Other Larynx Hypopharynx	25 34 9 13 1 2 9 8 4	35.2 47.9 12.7 18.3 1.4 2.8 12.7 11.3 5.6
HPV		
Positive Negative Not defined	19 13 39	26.8 18.3 54.9
HPV in relation to tumor origin		
Oropharynx HPV + HPV – not defined Other localization HPV + HPV – not defined	34 18 5 11 37 1 8 28	47.9 25.4 7 15.5 52.1 1.4 11.3 39.4
Alcohol		
No alcohol consumption Regular alcohol consumption Occasional alcohol consumption Former alcohol consumption Not defined	27 13 21 1 9	38 18.3 29.6 1.4 12.7
Smoker		
Never smoked Active smoker Former smoker Not defined	35 8 25 3	49.3 11.3 35.2 4.2

ECOG, Eastern Cooperative Oncology Group; HPV, human papillomavirus.

Written informed consent was obtained from all patients, allowing for the collection of their clinical data.

### 2.2 Treatment

Radiotherapeutic treatment included interstitial brachytherapy, postoperative (chemo)radiation, and definitive (chemo)radiation.

For definitive therapy, the standard dose prescription was 70 to 72 Gy for macroscopic tumor regions (2 Gy/fraction), 60 Gy for high-risk regions, and 50 Gy for low-risk regions. Platinum-based chemotherapy was indicated for all patients in the definitive setting.

In adjuvant treatment, the standard prescribed dose was 64 Gy (2 Gy/fx) to the former primary tumor region and in lymph node regions with extracapsular extension (ECE), 56 Gy (2 Gy/fx) in regions with lymph node metastases, and 50 Gy (2 Gy/fx) for elective nodal neck areas. Platinum-based chemotherapy was indicated in patients with resection margins ≥5 mm, ECE, or ≥3 lymph node metastases.

The standard treatment technique for external beam radiotherapy was volumetric-modulated arc therapy (VMAT) using 6-MV photons. The treatment position was supine with an immobilization mask. Treatment planning was based on 3-mm CT scans with contrast media unless contraindicated.

When indicated, the standard chemotherapy regimen was cisplatin-based with at least 200 mg/m^2^ body surface area (BSA) in total. Alternatively, carboplatin area under the curve (AUC) 1.5–2 weekly or equivalent was applied.

Follow-up examinations were conducted every 3 months after treatment, and typically every 6 months during the first 2 years, a CT scan of the neck and chest was performed. Regular follow-up visits were performed; parameters such as Eastern Cooperative Oncology Group (ECOG) performance status, diet and nutritional status, percutaneous endoscopic gastrostomy (PEG) use, xerostomia, dysphagia, voice changes and hoarseness, esophageal stenosis, trismus, and body mass index (BMI), were evaluated compared to baseline functions before the start of radiotherapy.

Detailed information about the follow-up is described in **Supplementary Table 2**.

### 2.3 Statistical analysis

Statistical analysis was performed using IBM SPSS version 26 (IBM Corporation, Armonk, NY, USA). Survival analyses were conducted using the Kaplan–Meier method. Overall survival and cumulative incidence of locoregional recurrence and distant metastases were calculated from the time of first diagnosis (defined as the date of biopsy or surgery of the primary tumor). For the statistical analysis, a dichotomous risk classification was used, including parameters such as smoker *vs.* non-smoker, regular alcohol consumption *vs.* no/occasional alcohol consumption, pre-conditions *vs.* no pre-conditions, cardiac pre-conditions *vs.* no cardiac pre-conditions, pulmonary pre-conditions *vs.* no pulmonary pre-conditions, nephrological pre-conditions *vs.* no nephrological pre-conditions, psychiatric pre-conditions *vs.* no psychiatric pre-conditions, stroke *vs.* no stroke, Barthel Index 85–100 *vs.* Barthel Index <85, ECOG 0–1 *vs.* ECOG ≥ 2, Charlson Comorbidity Index <5 points *vs.* Charlson Comorbidity Index ≥5 points, BMI < 25 *vs.* BMI > 25, Grading 1/2 *vs.* Grading 3, oropharynx *vs.* other tumor localization, standard therapy concept according to guidelines *vs.* different therapy concept (not according to guidelines), sole brachytherapy *vs.* definitive radiotherapy *vs.* adjuvant radiotherapy, definitive therapy intention *vs.* postoperative therapy intention, actual surgical therapy *vs.* non-surgical therapy, c/pT status 1/2 *vs.* c/pT status 3/4 c/pN status 1/2 *vs.* c/pN status 3/4, previous carcinoma *vs.* no previous carcinoma, and age < 80 *vs.* age ≥ 80.

Univariate statistical analysis was conducted using the log-rank test, and multivariate statistical analysis was performed using the Cox regression test. The general health factors including Barthel Index, ECOG performance status, and pre-conditions were compared for patients with a therapy according to guidelines and those with a deviation of a therapy according to guidelines using a chi-square test. A p-value of ≤0.05 was considered statistically significant.

## 3 Results

The median follow-up period was 18 months (95% CI, 1.0–128.0). The cumulative overall survival rates of this patient cohort after 1 year, 2 years, and 3 years were 83%, 79%, and 72%, respectively. During the follow-up period, 22 patients died. In four of these cases, the cause of death was confirmed to be tumor-related. A total of 28 patients were lost to follow-up (14 due to death and 14 due to unknown reasons).

Out of the 71 patients included, 13 (18%) underwent interstitial brachytherapy (seven postoperative and five definitive), 36 (51%) patients received definitive (chemo)radiotherapy, and 22 (30%) patients underwent postoperative (chemo)radiotherapy.

A total of 34 (48%) patients were treated with a standard therapy according to current guidelines.

A total of 45 patients received systemic therapy during treatment with 27 receiving platinum-based therapies and 18 receiving non-platinum (cetuximab)-based therapies. The average administered dose of cisplatin was 128.33 mg/m^2^ BSA (50% of patients switched to carboplatin due to toxicity). Patients receiving carboplatin-based chemotherapy were administered AUC1 (d1–5q28) or AUC 1.5–2.0 weekly (d1 q7).

**Figure 1:**
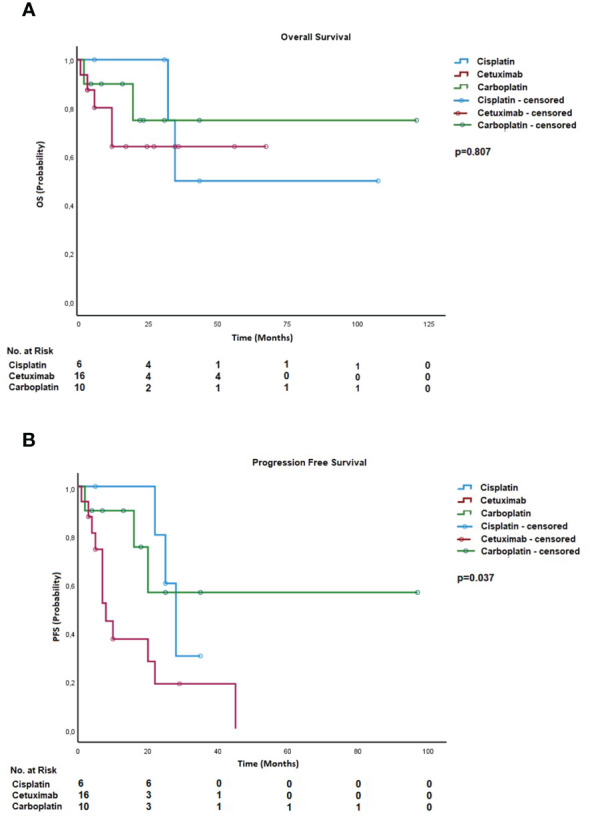
**(A)** Overall survival—systemic agent for definitive treatment. **(B)** Progression-free survival—systemic agent for definitive treatment.

**Figure 2:**
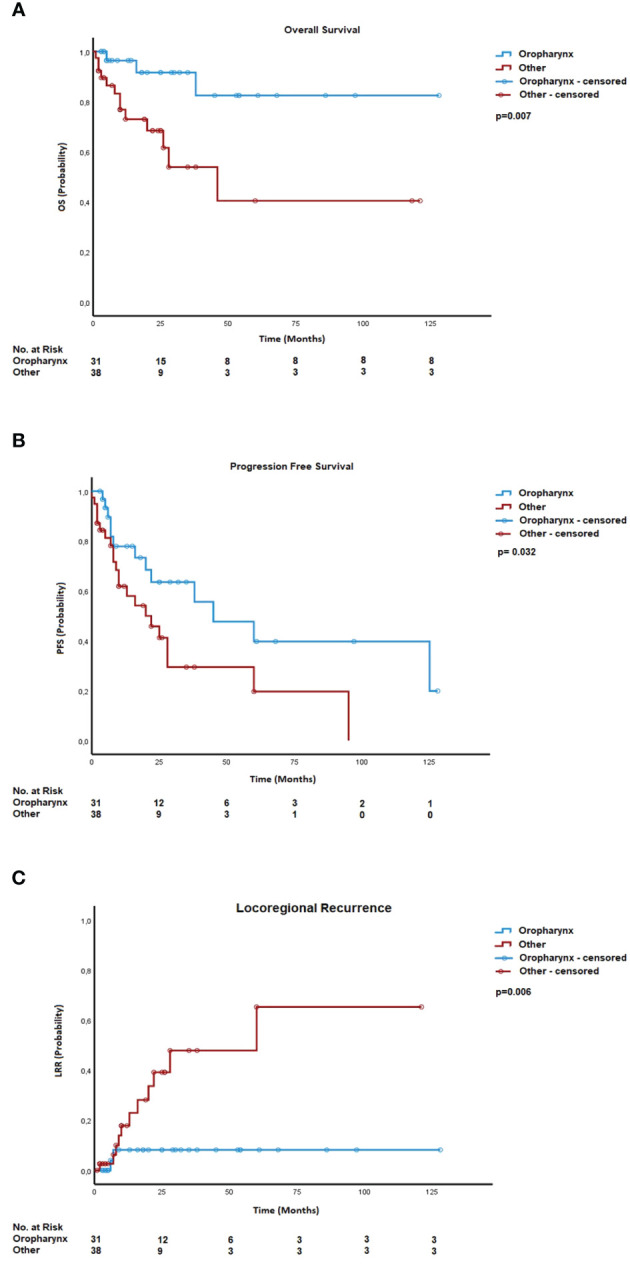
**(A)** Overall survival—localization of the tumor. **(B)** Progression-free survival—localization of the tumor. **(C)** Locoregional recurrences—localization of the tumor.

The analysis suggested that, in the case of definitive chemoradiation, carboplatin-based chemotherapy was associated with superior progression-free survival compared to cetuximab or cisplatin (**Figure 1B**), with absolute progression-free survival (PFS) being 60 months, 28 months, and 15 months, respectively (p = 0.037). However, no significant difference was observed regarding overall survival (**Figure 2A**). The small number of patients receiving chemotherapy in the adjuvant setting precluded statistical analysis. A total of 48 (67.6%) completed therapy as intended without treatment modification, and 23 (32.4%) patients discontinued radio- (n = 5) or chemotherapy (n = 18) or both. Further details about standard treatment and deviations are described in **Table 2**.

**Table 2:** Treatment characteristics.

Characteristics	No. of patients	%
Therapy intention		
Definitive brachytherapy Definitive external beam radiotherapy Definitive concurrent chemoradiation Adjuvant brachytherapy Adjuvant external beam radiotherapy Adjuvant concurrent chemoradiation	5 4 32 8 10 12	7.0 5.6 45.1 11.3 14.1 16.9
Standard therapy concept according to guidelines at the beginning of RT		
Yes No	34 37	47.9 52.1
Deviation of standard therapy concept		
Yes De-Escalation from cisplatin Reduction of external beam radiotherapy De-escalated systemic therapy and reduction external beam radiotherapy No systemic therapy Reduction of systemic therapy during treatment No	45 28 5 8 1 3 26	63.3 39.4 7.0 11.3 1.4 4.2 36.6
Dose external beam radiotherapy, Gy		
Median Range	64 18 – 72.6	
Discontinuation of external beam radiotherapy, n=58		
Yes No	5 53	8.6 91.4
Systemic therapy		
Yes No	45 26	63.4 36.6
Systemic therapies simultaneously to RT, n=45		
cisplatin/5-FU carboplatin/5-FU cisplatin mono carboplatin mono cetuximab paclitaxel & cisplatin	8 9 1 6 18 3	17.8 20.0 2.2 13.3 40.0 6.7
Dose reduction systemic therapy, n=45		
Yes No Not defined	7 33 5	15.6 73.3 11.1
Discontinuation of systemic therapy, n=45		
Yes No	18 27	40.0 60.0

RT, radiotherapy.

Univariate analysis identified several prognostic factors for overall survival (OS). OS rates for patients who did not consume alcohol were 92%, 85%, and 79% after 1 year, 2 years, and 3 years, respectively, compared to 54% after 1 and 2 years and 40% after 3 years for those who did consume alcohol. Factors such as non-alcohol consumption (p = 0.004), tumor localization (0.007), and combined treatment of surgery and postoperative therapy (p = 0.030) were associated with lower mortality risk and were included in multivariate testing via Cox regression. Non-alcohol consumption (p = 0.004), tumor localization (p = 0.026), and postoperative therapy (p = 0.008) remained significant predictors for overall survival.

Additionally, we performed univariate analysis on prognostic factors for PFS. Here, ECOG performance status (p = 0.031), Barthel Index (p = 0.027), tumor localization (0.032), c/pT status (p = 0.038) surgery, and postoperative radiotherapy (p = 0.041) were associated with lower risk for progression. Via Cox regression, only tumor localization (p = 0.011) and c/pT status (p = 0.038) remained significant for PFS.

The cumulative incidence of locoregional recurrences after 1 year, 2 years, and 3 years was 13%, 24%, and 27%, respectively. A significant impact was noted for the Barthel Index (p = 0.050) and tumor localizations (p = 0.006) on the occurrence of locoregional recurrence. For patients with a Barthel Index <85 points, the cumulative incidence of locoregional recurrences was 33% after 1 year and 66% after 2 years, whereas the cumulative incidence for patients with a Barthel Index >85 points was 9% after 1 year, 17% after 2 years, and 25% after 3 years.

For patients with an oropharyngeal tumor, the cumulative incidence of locoregional recurrences was 8% after 1 year, 2 years, and 3 years, while patients with a tumor on a different site revealed cumulative incidences of 18%, 39%, and 48% after 1 year, 2 years, and 3 years, respectively. Kaplan–Meier curves showed significant improvement in PFS, OS, and locoregional recurrence (LRR) for locations in the oropharynx (**Figures 1**, **2A–C**).

Multivariable analysis using Cox regression confirmed a significant impact for both tumor localization (p = 0.039) and Barthel Index (p = 0.028). For more detailed information about multivariate analysis, see **Table 3**.

**Table 3:** Multivariate Cox hazard models of prognostic factors for OS, PFS, and LRR.

	HR	95% CI	p-value
Prognostic factors for OS			
Alcohol consumption*	5.258	1.712–16.151	0.004#
Tumor localization*	4.950	1.214–20.187	0.026#
Postoperative therapy*	0.193	0.057–0.648	0.008#
Prognostic factors for PFS			
Tumor localization*	3.232	1.315–7.947	0.011#
c/pT status*	2.483	1.054–5.851	0.038#
Prognostic factors for LRR			
Barthel Index*	0.233	0.063–0.858	0.028#
Tumor localization*	5.408	1.090–26.843	0.039#

Univariate analysis was performed beforehand; only factors with an effect p-value of <0.1 in univariate Cox regression analysis were considered (*). # Final Cox regression model after backward selection. Only factors with p < 0.05 remained in the final model.

HR, hazard ratio; CI, confidence interval; OS, overall survival; PFS, progression-free survival; LRR, locoregional recurrence.

### 3.1 Safety

In terms of safety, we identified no treatment-related deaths. Concerning late toxicity, we were able to conduct regular follow-up examinations every 3 months in 43 patients (60.6%).

The most common treatment-related side effects in total consisted of dysphagia (60.5%) with 17 (39.9%) patients experiencing grade III dysphagia. Additionally, 30 (69.8%) patients described xerostomia, primarily grade I [17 patients (39.9%)].

A total of 22 (51.1%) patients were able to eat normally without supportive measures (PEG), and voice changes and hoarseness were only reported in 15 patients (34.9%). The treatment-related side effects varied slightly between the adjuvant and definitive treatment groups but were generally consistent in nature.

In adjuvant treatment, the most common treatment-related side effect was dysphagia (59.1%) with three patients experiencing grade III dysphagia. A total of 16 patients (72.7%) experienced xerostomia with the majority experiencing grade I (45.4%). PEG support was necessary for seven patients (33.3%). Voice changes were reported in nine patients (42.85%). In definitive treatment, reported toxicity was dysphagia of 57.9%, grade III in five patients (26.3%), xerostomia in 14 (73.68%), PEG support in 10 (42.1%), and voice changes in six (33.3%).

There were few cases of esophageal stenosis or trismus after therapy (four patients, 9.3% each).

For more detailed information about late side effects, see **Supplementary Table 3**.

## 4 Discussion

In this retrospective, single-center study, we evaluated outcomes and prognostic factors of old patients with primary head and neck squamous cell carcinoma without distant metastases at the time of initial diagnosis. The majority of existing studies concentrate on younger populations, leaving a gap for individuals aged 76 years and older, a demographic that is growing. In addition to that, generating prospective data is difficult to realize. Therefore, data concerning this age group are scarce, and to our knowledge, our study encompasses one of the largest cohorts to date for this specific patient group.

We found that a significant portion (52%) of older patients undergoing radio-oncological treatment for head and neck cancer had an excellent performance status (ECOG 0 and 1), and despite the diagnosis, cumulative overall survival was over 60% after 5 years. Additionally, the majority of patients in this age group were treated with definitive treatment concepts (57.7%), reflecting rather defensive surgical treatment due to perioperative risk factors and therefore apparent unfitness for surgical interventions.

Of the patients, 73% completed their therapy, indicating good treatment adherence, which is noteworthy given the expectation of lower compliance in this age group. This is especially true for radiotherapy alone (93%). Treatment modification or interruption was primarily due to reduced general health, patient request or tumor-related death, and modification of systemic therapy when applied. These modifications, driven by treatment-induced side effects, underline the importance of initial assessments of fragility and comorbidities to ensure treatment success. Most of the time, treatment decisions are made in the absence of personnel with geriatric specialization, which may facilitate the initial assessment of what treatment is bearable for older patients (6). Even though the personnel evaluating the ECOG/Barthel status is highly trained and is sorting the patients by well-thought-out and highly specific questionnaires, false assessment in this process may lead to under-treatment in HNSCC patients, which occurs frequently and can be prevented by a multidisciplinary approach (7). In this context, we were able to show that in patients with indication for chemoradiation, only 54% (n = 24) were treated with platinum-based chemotherapy. Most of the patients deemed platinum-unfit were treated with EGFR antibody cetuximab instead.

This consideration plays a crucial role in interpreting treatment efficacy. The choice of initial treatment, such as combined chemoradiation *versus* radiotherapy alone, is largely determined by the patient’s overall health status and existing comorbidities. Patients who are candidates for combined therapy may inherently have a better OS to begin with. This potential bias must be acknowledged and factored into any analysis of treatment outcomes to ensure an accurate understanding of the efficacy of different treatment modalities.

Comparing all systemic agents in the definitive setting, patients treated with carboplatin appeared to positively influence PFS, but no significant change in OS was evident. The low number of patients receiving chemotherapy in adjuvant treatment did not allow for statistical analysis. Certainly, those results need to be interpreted cautiously due to small and unbalanced group sizes. Also, patients receiving cisplatin often did not receive the full dose (five out of 11, mean cisplatin dose 128.33 mg/m^2^ BSA overall), and systemic therapy needed to be aborted due to complications. Only in some cases did the switch to carboplatin-based therapy achieve equivalent doses of approximately 200 mg/m^2^ BSA in total. This stresses the fact that initial treatment decisions on the right choice of systemic agent should be made in the context of possible complications in old patients in order to complete therapy as intended.

In accordance with other retrospective studies including patients of older age, the findings of this study suggest that the overall survival does not correlate with the chronological age but rather with other characteristics like tumor localization and treatment intent of patients (8, 9).

Contrary to expectations, the ECOG performance status, Barthel Index, and Charlson Comorbidity Index did not show a correlation with survival outcomes. In univariate analyses, a lower Barthel Index was associated with decreased PFS, but this association did not hold significance when subjected to backward testing. This suggests that the impact of a low Barthel Index on outcomes may not be solely due to patient frailty before treatment but could also be influenced by the subsequent choice to de-escalate therapy, which in turn may lead to higher rates of LRR observed in initial testing. The inability to maintain the significance of these factors as predictors upon further analysis could be attributed to the overall higher burden of comorbidities (Charlson score >2) in our elderly cohort, which may inherently affect OS rates. This highlights the complex interplay between patient functional status, treatment decisions, and the inherent risk posed by comorbid conditions in influencing outcomes for older patients with head and neck cancer.

Rather than performance status, tumor location in the oropharynx had significantly better OS, PFS, and LRR. This has to be interpreted in the context of oropharyngeal tumors showing a higher association with human papillomavirus (HPV) (8–10). Due to a higher treatment response rate on chemotherapy as well as radiotherapy, HPV-positive tumors have better outcomes than HPV-negative tumors (8–10). Despite a large meta-analysis showing no benefit for chemoradiation in older patients, systemic therapy should not be entirely neglected in these tumor locations (11).

Our study’s findings support the notion that therapy decisions should consider clinical and prognostic factors over patient age alone (1, 4, 10, 12–16). In accordance with Roden et al., who observed patients with HNSCC undergoing surgery, the findings of this study also suggest no significant difference between guideline-based treatment and treatment not according to guidelines (17), showing that treatment modifications are acceptable to ensure adherence and outcomes.

Approximately 40% of the patients were lost to follow-up. Future efforts should aim for closer monitoring of patients with reduced ECOG performance status to address tumor or therapy-related side effects and to improve quality of life. Most side effects were generally in line with those experienced by younger cohorts and ranged mainly within expectations. Importantly and along with most of the analyses of older age groups, no treatment-related deaths were reported, indicating that definitive or adjuvant (chemo)radiation is a feasible treatment method.

In spite of adding to the growing body of knowledge in the treatment of old patients with HNSCC, our study has certain limitations. First, due to its retrospective nature, there is a potential for miscoding.

In order to have a better comparability, we furthermore only included patients with cM0 status in this study. Patients with cM1 have a presumed worse therapy outcome; thus, treatment options in patients with cM1 differ from those with cM0. As those with cM1 were excluded, we cannot make any statements about that patient cohort.

As our study was performed by the Department of Radiation Oncology, our special attention fell onto the patients receiving radiotherapy—solely or in combination with other standardized procedures such as chemotherapy or surgical therapy. As a result, patients who received only surgical treatment were not included in our analysis.

Additionally, we encountered a significant loss in follow-up shortly after treatment began, which hindered our ability to document late side effects for some patients. Furthermore, our data lack immunohistochemical information regarding HPV status for some patients, a factor that could significantly affect treatment outcomes especially when combining radiotherapy with chemotherapy (8–10). Conclusions on the efficacy of systemic agents without stratifying for HPV status should be approached with caution. Similarly, the analysis lacked more detailed information on patient characteristics, such as smoking habits, which could explain why this significant risk factor was omitted from the survival analysis. This gap in data underlines the need for comprehensive patient tracking and the inclusion of detailed patient characteristics in future research to better understand its influence on the efficacy of treatments for head and neck cancers.

## 5 Conclusion

Older patients represent a vulnerable patient group, presenting unique characteristics that often require tailored treatment approaches. Our analysis demonstrates that deviations from standard treatment did not worsen outcomes in old patients. With our analysis, we were able to show that treatment of older-age HNSCC patients is feasible and can positively impact LRR, OS, and PFS while maintaining acceptable toxicity. Certainly, more data are needed in order to refine and adjust current treatment strategies specifically for this patient demographic.

## Data availability statement

The raw data supporting the conclusions of this article will be made available by the authors, without undue reservation.

## Ethics statement

Ethical approval was not required for the study involving humans in accordance with the local legislation and institutional requirements. Written informed consent to participate in this study was not required from the participants or the participants’ legal guardians/next of kin in accordance with the national legislation and the institutional requirements.

## Author contributions

L-SW: Writing – review & editing, Conceptualization, Data curation, Formal analysis, Funding acquisition, Investigation, Methodology, Project administration, Resources, Software, Supervision, Validation, Visualization, Writing – original draft. MH: Conceptualization, Data curation, Formal analysis, Funding acquisition, Investigation, Methodology, Project administration, Resources, Software, Supervision, Validation, Visualization, Writing – original draft, Writing – review & editing. SS: Data curation, Formal analysis, Funding acquisition, Investigation, Methodology, Project administration, Resources, Software, Supervision, Validation, Visualization, Writing – original draft, Writing – review & editing, Conceptualization. DH: Writing – original draft, Writing – review & editing. FP: Writing – original draft, Writing – review & editing. SM: Writing – original draft, Writing – review & editing. HI: Writing – original draft, Writing – review & editing. MK: Writing – original draft, Writing – review & editing. RF: Writing – original draft, Writing – review & editing. PS: Conceptualization, Data curation, Formal analysis, Funding acquisition, Investigation, Methodology, Project administration, Resources, Software, Supervision, Validation, Visualization, Writing – original draft, Writing – review & editing.
