# Supplementary material for: Treatment with (chemo)-radiation in old patients (≥76 years of age) with newly diagnosed non-metastatic squamous cell cancer of the head and neck region: real-world data from a tertiary referral center
Source: Front Oncol. 2024 Apr 25;14:1382405. doi: 10.3389/fonc.2024.1382405 (PMC11079188; doi:10.3389/fonc.2024.1382405)
Supplement: Supplementary file 1 [file Table_1.docx]

**Supp. TABLE 1 Detailed Patient Characteristics**

| Characteristics | No. of patients | % |
| --- | --- | --- |
| Primary tumor site  Oral cavity  Oropharynx  Base of tongue  Tonsil  Uvula  Palate  Other  Larynx  Hypopharynx | 25  34  9  13  1  2  9  8  4 | 35.2  47.9  12.7  18.3  1.4  2.8  12.7  11.3  5.6 |
| Therapy intention  Postoperative  Definitive | 30  41 | 42.3  57.7 |
| cT classification, n=39  T1  T2  T3  T4 | 3  17  9  10 | 7.7  43.6  23.1  25.6 |
| cN classification, n=39  N0  N1  N2  N2a  N2b  N2c  N3 | 15  9  13    7  6  2 | 38.5  23.1  33.3  17.9  15.4  5.1 |
| pT classification, n=32  T1  T2  T3  T4  T4a | 10  9  6  7  6 | 31.3  28.1  18.8  21.9  18.8 |
| pN classification, n=32  N0  N1  N2  N2a  N2b  N2c  N3 | 17  2  12  4  5  3  1 | 53.1  6.3  37.5  12.5  15.6  9.4  3.1 |
| Perinodal spread, n=32  Yes  No | 6  26 | 18.75 81.25 |
| Lymphangiosis, n=32  L0  L1  Not defined | 27  4  1 | 84.4  12.5  3.1 |
| Hemangiosis, n=32  V0  V1  Not defined | 30  1  1 | 93.8  3.1  3.1 |
| Perineural spread, n=32  Pn0  Pn1  Not defined | 28  3  1 | 87.5  9.4  3.1 |
| Resection status, n=32  R0  R1  R2  Not defined | 28  2  1  1 | 87.5  6.3  3.1  3.1 |
| Grading  G1  G2  G3 | 5  20  46 | 7.0  28.2  64.8 |
| HPV  Positive  Negative  Not defined | 19  13  39 | 26.8  18.3  54.9 |
| Total removed lymph nodes, n  Median  Range | 23.5  11 – 91 |  |
| No. of affected lymph nodes, n  Median  Range | 0  0 – 33 |  |

**Supp. TABLE 2 - Follow up**

| Characteristics | No. of patients | % |
| --- | --- | --- |
| Local recurrence  Yes  No | 13  58 | 18.3  81.7 |
| Localization of local recurrence, n=13  Primary tumor site  Cervical lymph nodes | 11  2 | 84.6  15.4 |
| Remote metastases  Yes  No | 6  65 | 8.5  91.5 |
| Localization of remote metastases, n=6  Lungs  Mediastinal lymph nodes | 3  3 | 50  50 |
| Age at tumor recurrence, y  Median  Range | 82  78 – 86 |  |
| Secondary tumor  Yes  No | 7  64 | 9.9  90.1 |
| Localization of secondary tumor, n=7  Oral cavity  Oropharynx  Colon  Prostate | 1  1  2  3 | 14.3  14.3  28.6  42.9 |
| Age at secondary tumor, y  Median  Range | 78  76 – 87 |  |

**Supp. Table 3 - Follow up – Side effects**

| Characteristics | No. of patients | % |
| --- | --- | --- |
| Follow-up  Yes  No | 43  28 | 60.6  39.4 |
| Median Follow-up, n=43 | Every 3 months |  |
| Tumor-related death, n=71  Yes  Cause of death uncertain  Patient is alive/Death unknown | 4  18  49 | 5.6  25.4  69.0 |
| ECOG – latest follow-up, n=43  Asymptomatic (0)  Symptomatic but completely ambulatory (1)  Symptomatic, <50% in bed during the day (2)  Symptomatic, >50% in bed, not bedbound (3)  Bedbound (4)  Death (5)  Not defined | 17  15  5  1  5 | 39.5  34.9  11.6  2.3  11.6 |
| Diet and nutrition after treatment – follow-up, n=43  Normal  Soft/pureed  Additional via PEG  Mostly via PEG  Solely via PEG  Not defined | 22  1  9  2  6  3 | 51.1  2.3  20.9  4.7  14.0  7.0 |
| Xerostomia – latest follow-up, n=43  Yes  Grade 1  Grade 2  Grade 3  No  Not defined | 30  15  9  6  11  2 | 69.8  34.9  20.9  14.0  25.6  4.7 |
| Dysphagia – latest follow-up, n=43  Yes  Grade 1  Grade 2  Grade 3  No  Not defined | 26  4  5  17  15  2 | 60.5  9.3  11.6  39.5  34.9  4.7 |
| Voice changes and hoarseness – latest follow-up, n=43  Yes  Grade 1  Grade 2  Grade 3  Grade 4  No  Not defined | 15  7  4  2  2  27  1 | 34.9  16.3  9.3  4.7  4.7  62.8  2.3 |
| Esophageal stenosis – latest follow-up, n=43  Yes  Grade 1  Grade 2  Grade 3  No  Not defined | 4  2  2    37  2 | 9.3  4.6  4.7  86.0  4.7 |
| Trismus – latest follow-up, n=43  Yes  Grade 1  Grade 2  Grade 3  No  Not defined | 4  2  1  1  38  1 | 9.3  4.7  2.3  2.3  88.4  2.3 |
| BMI after therapy – latest follow-up, n=43  Underweight <18.5  Normal 18.5-24.9  Overweight 25-29.9  Grade I obesity (moderate) 30-34.9  Grade II obesity (severe) 35-39.9  Grade III obesity (clinically) >40.0  Not defined | 3  24  12  1  3 | 7.0  55.8  27.9  2.3  7.0 |
